# Fashions in Bibles

**Published:** 1885-01

**Authors:** 


### FASHIONS IN BIBLES.

Fashion enters into the Bible business as much perhaps as into the matter of church dresses, and the styles have never been as numerous and beautiful as now. When Moody and Sanky were in the zenith of their fame in this country, it was observed that Mr. Moody used a peculiar Bible bound in limp covers, the leather of which projected aad turned down to protect the gilt edges of the book. It was a Bible published by a comparatively small London house, and had as an appendix a condensed concordance and much general and chronological information, such as a description of all animals and mountains mentioned in the Bible. Although it was so valuable that it retailed as high as $14, every Bible student was bound to have one as soon as it was learned that Mr. Moody used one of them, and the publishers made a fortune out of them, and scornfully rejected overtures from a rich Bible house enjoying the title of the Queen's publishers. Then the latter house employed Bible students who wrote matter for an appendix as exhaustible as that of the Bible used by Mr. Moody, and the firm have since sold a quarter of a million of them. The revised New Testament had an immense sale for a while, and then it fell flat, and very few are now sold, the old version being demanded. It took fifty years to introduce the King James version of the Bible. The English are underselling the Americans in small Bibles and prayer books, but the Americans have a monopoly in the sale of large family and pulpit Bibles. Very few illustrated Bibles are demanded, the Dore Bible being the most popular. The Turkey morocco that is used in bindings is technically called Persian leather. It is goatskin tanned in Persia. It is easily worked and embossed in fifty or more different styles. There is an imitation in color of leopard skin, and there is a cover closely resembling the shell of the armadillo. Another style is a close imitation of ivory, and another is bound in genuine antique crocodile, a small Bible thus encased selling at $15. Nile Persian, Indian Persian, and renaissance calf are among the other fashionable new bindings. Episcopal prayer books and hymnals are bound separately and enclosed together in a sort of portfolio, with an ingeniously arranged handle. Publishers are compelled by the rules of the Church to bind them apart, because they have to pay a royalty of 10 per cent. on the hymnal, which money goes to the fund for the support of indigent pastors. Catholic prayer books are a profitable branch of the trade, because there are over 700 versions with different titles, although all are the same in substance. —*New York Sun.*

